# Seminal papers in urology: two-year outcomes of Sacral Neuromodulation Versus OnabotulinumtoxinA for refractory urgency urinary incontinence: a Randomized Trial

**DOI:** 10.1186/s12894-023-01385-5

**Published:** 2024-01-16

**Authors:** Haidar Hadri bin Abd Wahab, Michael O’Callaghan

**Affiliations:** 1 Urology Unit, Flinders Medical Centre, Bedford Park, Australia; 2 College of Medicine and Public Health, Flinders University, Adelaide, Australia; 3 Discipline of Medicine, The University of Adelaide, Adelaide, Australia

**Keywords:** Urinary incontinence, Randomized controlled trial, Sacral Neuromodulation, OnabotulinumtoxinA

## Abstract

In this critical review, we explore the study design, strengths and limitations of the paper: “Two-Year Outcomes of Sacral Neuromodulation Versus OnabotulinumtoxinA for Refractory Urgency Urinary Incontinence: A Randomized Trial.” The paper reports 24 month follow-up data of the landmark ROSETTA trial. This multi-centre, open-labelled parallel randomised trial allocated females 1:1 to receive Sacral Neuromodulation (SNM) or OnabotulinumtoxinA(BTX) 200 units (U). The primary outcome was change in mean daily urinary urgency incontinence episodes (UUIE) over 24 months. The study did not demonstrate a difference between treatments (-3.88 vs. -3.50 episodes per day), however women treated with BTX were more satisfied; but reported higher rates of UTI. The two treatments provide comparable third-line treatment options for patients with refractory urgency urinary incontinence.

## Background

Urgency urinary incontinence (UUI) is defined as involuntary urine leakage associated with a sudden compelling desire to void, with a prevalence of up to 30.3% for women and 22.8% for men worldwide [1]. Current recommendations by the European Association of Urology (EUA) [2] and American Urological Association (AUA) [3] guidelines include behavioural therapies as first-line therapy and pharmacological interventions as second-line therapy. Third-line therapies such as OnabotulinumtoxinA (BTX) and sacral neuromodulation (SNM) are recommended for patients with UUI refractory to behavioural and pharmacological management. SNM has cure rates of 15% at five years and 17% at 10 years, while BTX’s cure rates ranges from 15.9 to 50.9% at three months and 31.1% at six months for UUI [4]. The ROSETTA trial (Refractory Overactive Bladder: Sacral Neuromodulation versus Botulinum Toxin Assessment) first reported outcomes in 2016, showing a statistically significant daily improvement of UUI in BTX compared to SNX at six months [5].

## Study characteristics

Amundsen et al. subsequently report 24 month follow-up data in the landmark paper: “Two-Year Outcomes of Sacral Neuromodulation Versus OnabotulinumtoxinA for Refractory Urgency Urinary Incontinence: A Randomized Trial” [6], published 2018. The trial compares BTX and SNM with outcomes of UUI episodes (UUIE), diary results, quality of life measures (QOL) and adverse events (AE) results in women. The initial study was a multi-centre, open-labelled parallel randomised trial where participants were randomised 1:1 to receive SNM or BTX 200 units (U). Enrolment of participants followed the published ROSETTA trial study protocol, at nine US Obstetrics and Gynaecology and Urology departments across different states and demographic areas to maximise generalisability [7]. Randomisation was done using permuted blocks with a fixed block size known only to the data collection centre, and implementation of the random allocation sequence was done using a web-based application [7]. Participants were then stratified by age (< 65 and ≥ 65 years) and study site. The eligibility criteria consisted of participants who had six or more UUIE and refractory to first-line and second-line therapies. The intervention arms also included alternative treatments for those who failed to respond to their own respective initial intervention. There were 386 women who were randomised into two groups, 192 to receive BTX and 194 to receive SNM. This exceeded the calculated sample of 158 patients per group allowing 20% loss to follow-up, α = 5%, SD 6.0, 80% power to detect − 2.0 UUIE per day. Clinical responders (CRs) to initial SNM lead placement who had more than 50% reduction in UUIE after proceeding to Stage II pulse generator placement, while non-responders were allowed medication and could receive BTX therapy after six months. Similarly, non-responders to BTX injection were also allowed medication and could receive SNM therapy after six months. In both intervention arms, additional treatment, including reprogramming, surgical revision and device removal, was offered if the patient’s Global Symptom Control (PGSC) scores were 1 or 2. Meanwhile, participants requiring prolonged clean intermittent catheterisation (CIC) post BTX injections had reduced dose of BTX and was offered a third injection [6].

The primary endpoint was a change in mean daily UUIE collected at baseline and over the next 24 months. Secondary endpoints were no UUIE, ≥ 75% and ≥ 50% UUIE reduction from diary results, Quality of Life measurements using Overactive Bladder Questionnaire Short Form (OAB-SATq), Urinary Distress Inventory short form (UDI-SF), Incontinence Impact Questionnaire and the Sandvik Incontinence Severity index and adverse events.

A linear mixed model was used for the primary analysis of continuous UI measure where the monthly change from baseline in mean UUIE per day was used as an outcome. Treatment differences in binary diary and QOL outcomes were evaluated using analogous generalised linear models based on Poisson regression. AE measurements across treatment arms were compared using Fisher’s exact tests.

## Summary of outcomes

At six months, participants who received BTX (*n* = 159) reported as more likely to demonstrate complete UUI resolution (treatment difference = − 18%,95% CI = − 29 - -6; *P* < 0.001) and ≥ 75 UUIE reduction (treatment difference = − 20%; 95% CI = − 31 - -8; *p* = 0.001). However, there was no difference in mean UUIE decrease at 24 months (− 3.88 vs. − 3.50 episodes/day; mean difference = 0.38; 95% CI = -0.14–0.89; *p* = 0.2) and there were similar rates of complete resolution (5%) and > 75% reduction (22% for BTX and 21% for SNM).

Participants who requested additional medications (BTX 21% [34/159], SNM 21% [29/159], *p* = 0.7) or alternative trial therapy off protocol (BTX 6% [34/159], SNM 5% [29/159]) were comparable based on QOL measures.

More than half of BTX participants (72%, [115/159]) requested a second injection, where 88%(101/115) received 200 U, of which 6%(6/115) required clean intermittent catheterization (CIC). Any participants who required CIC > six months had reduced dose of BTX injections. 12% (14/115) were dose-reduced from 200 U to 100 U and 21% (3/14) required CIC after 100U. The median CIC duration was 29 days (IQR 17–56) across nine participants with 24% (45/189) requiring CIC at any point within 24 months.

Furthermore, 48%(55/115) requested a third injection with a median interval of 273 days (IQR 224–350) between the second and third injections. 58% of the SNM group required programming, with only 17% requiring ≥ 3 reprogramming. Overall, there were higher rates of UTI in the BTX group compared to SNM group with 36% vs. 15% (*p* = < 0.001) at 1–6 months, 22% vs. 12% (*p* = 0.012) at 7–12 months and 18% vs. 8% (*p* = 0.006).

Overall, the data from this study showed comparable outcomes which supports the current guidelines available for UUI management [2, 3].

## Assessment of evidence

Using the Cochrane risk-of-bias tool version 2 to assess this study, we classified the paper as ‘high risk’ of bias. This included domain scores of ‘low’ for risk of bias arising from the randomisation process; ‘some concerns’ due to deviation from intended interventions; ‘high risk’ due to missing outcome data (no evidence that the result is not biased through alternate analysis); ‘some concerns’ due to measurement of the outcome; and ‘some concerns’ due to selection of the reported result [8]. Many of these concerns are difficult to eliminate in the context of a surgical trial where patients are (appropriately) rating their own quality of life and symptoms.

While long term outcomes are important, this is a secondary analysis completed at 24 months post intervention, with the main trial being planned and powered for six month outcomes.

## Future research

The ROSETTA trial was also designed to include economic evaluation, with a separate analysis showing that SNM costs over two years were significantly higher than BTX ($35,680 [95% CI 33,920 − 37,440] vs. $7,460 [95% CI 5,780-9,150], *p* < 0.01) [9].

Since its publication in 2018, this has been cited in a systemic review and meta-analysis, being the only RCT identified to compare SNM and BTX in 2021 [10]. The synthesised evidence showed a consistent picture of SNM and BTX having substantially the same effect on incontinence events, with BTX having a higher UTI rate, and SNM having a higher cost. This highlights the need for clinically effective interventions that have both low cost and low levels of adverse events, together with the need for more randomised evidence in this area. A candidate may be offered posterior nerve stimulation (PTNS) [11] prior to being offered SNM or BTX for managing UI, which offers substantial cost savings [12]. Further studies comparing SNM, BTX with PTNS and other surgical options for UI such as bladder augmentation or urinary diversion may provide valuable insight in terms of costs, morbidity and mortality.

## Data Availability

Not applicable.
